# The Impact of the COVID-19 Pandemic on the Quality of Educational Process: A Student Survey

**DOI:** 10.3390/ijerph17217770

**Published:** 2020-10-23

**Authors:** Maria-Crina Radu, Carol Schnakovszky, Eugen Herghelegiu, Vlad-Andrei Ciubotariu, Ion Cristea

**Affiliations:** Department of Industrial System Engineering and Management, Faculty of Engineering, “Vasile Alecsandri” University of Bacau, Calea Marasesti 157, 600115 Bacau, Romania; scarol@ub.ro (C.S.); eugen.herghelegiu@ub.ro (E.H.); vlad.ciubotariu@ub.ro (V.-A.C.); icristea@ub.ro (I.C.)

**Keywords:** COVID-19 pandemic, lockdown period, quality of educational process, online platforms, student survey

## Abstract

The paper presents the results of a students’ survey carried out at “Vasile Alecsandri” University of Bacau, Romania, on the quality of educational process on online platforms in the context of the COVID-19 pandemic. The study was addressed to students from the Faculty of Engineering and the Faculty of Physical Education and Sports. The results of survey highlighted that most of students were satisfied with the measures taken by the university during the lockdown period and the way the teaching—learning-assessment process took place. However, some negative aspects were reported as: lack of an adequate infrastructure for some students, less effective teacher-student communication and interaction, impossibility of performing practical applications, lack of socialization, lack of learning motivation, less objective examination (e.g., possibility of cheating), possibility of physical and mental health degradation (e.g., too much time spent in front of screens, installation of a sedentary lifestyle). Consequently, for the new academic year, effective, and efficient measures must be implemented by the management of the university to remove, as much as possible, these negative issues and to improve the performance of online educational process.

## 1. Introduction

Education is “a fundamental human right, a global common good and a primary driver of progress across all the 17 Sustainable Development Goals (SDG) of the 2030 Agenda as a bedrock of just, equal, inclusive, peaceful societies” [1]. The COVID-19 pandemic, labelled as a “black swan” event [2], “catastrophic calamity” [3] and compared to the World War II in terms of economic and societal consequences [4,5], has caused the largest disruption of education in history, and has already had a near universal impact on learners and teachers around the world [6,7].

In the higher education sector, universities have been forced to close the doors in response to the growing coronavirus outbreak and, where the IT infrastructure allowed, switch classes to online learning to keep students’ retention and maintain access to learning [8]. According to a survey carried out by the International Association of Universities (IAU) from 25 March to 17 April 2020, two-thirds of higher education institutions were able to move teaching online while one third could not [9]. A solution to continue teaching and learning activities was offered by the online platforms [10,11,12,13]. However, even where the necessary infrastructure existed, the immediate challenge generated by the lockdown was to ensure clear and effective communication streams with staff and students. Teachers did not necessarily have the appropriate skills so that they could suddenly and easily shift from face-to-face to online teaching and often resulted in “learning by doing” or imitating the face-to-face approach, which, in turn, may not guarantee the same level of quality of the educational process [14,15]. According to [16], a temporary shift to an alternate teaching mode due to crisis circumstances (e.g., the COVID-19 pandemic) cannot be equated with a well-planned and designed online education process and, in our opinion, even less with face-to-face learning. Besides, technical challenges can determine senior teachers with a long institutional tenure to leave the system, which could lead to the loss of rich organizational experience [17]. A great burden was placed also on students who suddenly had to possess a variety of skills, competencies, and resources [18]. These issues have impacted the emotional, psychological, and social well-being of both teachers and students all around the world. For instance, a study focusing on the psychological impact of COVID-19 at a Spanish university revealed moderate to extremely severe scores of anxiety, depression, and stress for students and lower scores for the university’s staff [19]. Higher anxiety levels due to COVID-19 related stressors was also reported for Chinese medical college students [20]. Stress, anxiety, and worries about coronavirus contamination and change of mental health were also highlighted in a quantitative and qualitative study carried out on students from a public university in the United States [21]. Moreover, students with less opportunities (e.g., lack of digital or inappropriate equipment, lack of Internet or slow connection to Internet networks) and poor digital skills, were likely to suffer more because of the online instruction, leading to an intensification of the existing inequities [22,23]. In [21], it is shown that students who did not use educational technologies had a lower perception of self-efficacity, which in turn led to a lower cognitive engagement. A similar idea was promoted in [24], which showed that the attitude of students towards educational technology directly affected their learning process; a negative attitude negatively impacted their academic performance.

The aim of this paper was to present the results of a students’ survey carried out at “Vasile Alecsandri” University of Bacau (UBc), Romania, on the quality of teaching–learning assessment process on online platforms, in the context of the COVID-19 pandemic. 

## 2. Materials and Methods 

### 2.1. The External and Internal Context of the UBc

Considering the evolution of the international epidemiological situation caused by the spread of SARS-CoV-2 coronavirus as well as the declaration of the “Pandemic” by the World Health Organization (WHO) on 11 March 2020 the emergency state was declared in Romania, starting with 16 March 2020 [25]. On 11 March 2020, the Administrative Council (AC) of UBc decided to suspend teaching and tutorial activities, between 13 and 22 March 2020. With the establishment of the emergency state, the University’s AC decided to extend the suspension period until 29 March 2020, and to do the teaching activities online. All the teachers received the contact details from the secretariat of each faculty of the group leaders from each study programme to be able to communicate easily with the students. 

Rapid solutions for the online courses had to be provided in order to avoid a negative impact on the teaching-learning process. In the beginning, the communication with students was done by e-mail, on different platforms, applications (e.g., Zoom, Skype, WhatsApp) and social networks, or by phone. Meanwhile, the university’s management analysed several online platforms in order to choose the most convenient one. A comparative analysis was carried out, considering different criteria such as: the maximum number of participants in the free/full version, privacy, security and end-to-end (E2E) encryption, the possibility to make recordings, exclusive features and applications Integration (APPS Integration), the use of the whiteboard for teaching (Table 1) [26,27,28]. As a result of the analysis, the Microsoft Teams platform was chosen and implemented, based on the existing infrastructure. Starting on 15 April 2020, the platform was fully functional and the switch to the online teaching-learning process was made. Tutorials were created on how to use the platform, both for teachers and students. Teachers who had used until then other communication means for the online teaching activities, could keep them further. However, the exams (including those for the completion of studies) were taken only on the Microsoft Teams platform, according to the initial planning, and only the re-examinations were postponed for the September 2020 session, except for those related to the final year of studies. 

### 2.2. The Objectives of the Survey

The classical educational process has been severely disrupted by the COVID-19 pandemic and by administrative measures taken at the national, regional, and local levels to prevent the spread of the coronavirus infection. Thus, the UBc’s academic staff was taken by surprise in terms of digital teaching, online knowledge transmission, and the use of appropriate technologies to online activities.

The main objective of the survey was to inspect the perception of UBc students regarding the online education, given the fact that, up to this level (i.e., bachelor’s and master’s degree), they have never faced this form of education. It is worth mentioning that, in Romania, and particularly at the UBc, the classic form of education is face-to-face education. Likewise, the study did not want to confirm or deny certain hypotheses; it was rather intended to be an analysis of the University’s situation in terms of online education through the feedback of their own students, so as to be a starting point for immediate measures to be implemented at the beginning of the new academic year and for further research related to the development of online teaching methods and tools. 

The specific derived objectives we pursued were:

- determining the adequacy of the existing infrastructure and the implemented online platform;
- identifying the degree to which teachers cope with this way of teaching/evaluation;
- identifying the negative aspects reported by students in order to offer future solutions to remedy them, if the COVID-19 pandemic persists or will be repeated over time.

### 2.3. The Study Sample

The target group consisted of students from two faculties: Faculty of Engineering (FE), bachelor and master’s degree, and Faculty of Movements, Sports and Health Sciences (FPSE), bachelor’s degree. The two faculties were chosen due to their specificity, namely many disciplines imply the use of laboratories. A call for participation was also shared through WhatsApp application, by using the existing groups, created for teacher-student communication at the two faculties. The calculation of a correct response rate was not possible because of this channel of communication. Data collection was gathered for two weeks.

135 students (99 students from FE and 36 students from FPSE, Table 2) responded to the survey and key trends were identified across the responses concerning their perception over the quality of the educational process on online platforms.

### 2.4. The Design of the Survey

The basic method used in conducting the study was the survey method, which used a questionnaire as a tool. An anonymous online survey assessing the students’ opinion on the quality of the educational process on online platforms was created using the Google Forms online application. The survey consisted in 12 questions (see Supplemental Content—The questionnaire in Supplementary Materials) that had as a starting point the public consultation of the European Commission on Digital Education Action Plan. It is worth mentioning that the current survey is not connected to the public consultation in question, and it is neither commissioned nor sponsored by the Commission; the survey period and the sample of respondents are different from the Commission’s and thus the results are distinct and without prejudice to the outcome of the abovementioned consultation. 

Nine questions in the survey were closed questions; students had to choose between multiple options or rank-order them, using five levels ordinal scales. There were also three open-ended questions, which gave students the opportunity to indicate examples of tools, including digital platforms they found particularly useful for online learning during the lockdown period as well as the advantages and the disadvantages of online examination.

Students’ responses were automatically collected and statistically processed by the Google Forms application. Data were then imported into the Excel program (Office 365) for a clearer graphical representation of them, which facilitated their analysis and interpretation. The answers to the open-ended questions were categorized on the criterion of similarity and the most frequently reported aspects are presented in the manuscript.

## 3. Results

**Figure 1:**
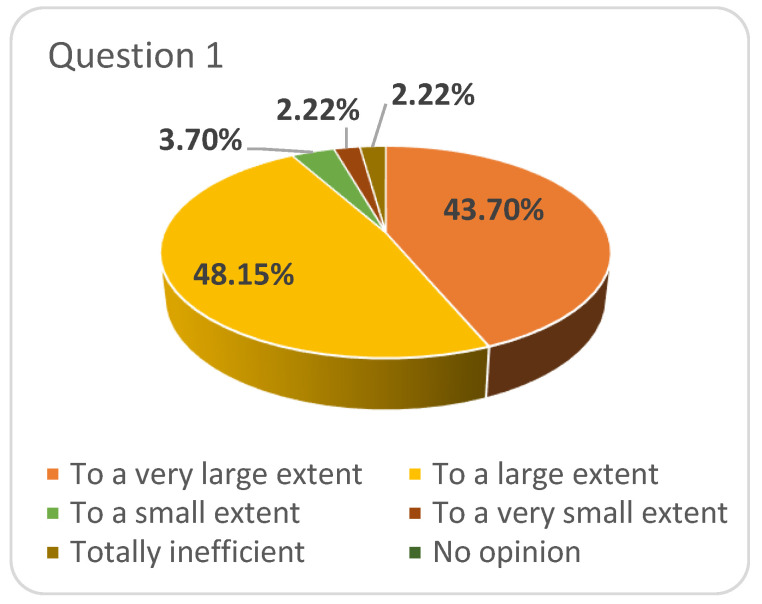
Respondents’ answers to question 1: To what extent do you consider that the measures taken by “Vasile Alecsandri” University of Bacau during the COVID-19 pandemic to ensure the continuity of the educational process (teaching–learning–assessment) were sufficient and effective.

The majority of the respondents considered that the measures taken by “Vasile Alecsandri” University of Bacau during the COVID-19 pandemic to ensure the continuity of the educational process (question 1) were sufficient and effective (Figure 1) and only few of them (4 students from FPSE first year and 2 students from FE, third year) stated the opposite.

The items used to assess the online teaching, learning and assessment/examination experience during the COVID-19 pandemic (question 2) were ranked as “good” and “very good” by most of the students (Table 3). However, special attention should be paid to those students who did not give these scores because they may belong to a vulnerable category that requires the implementation by the university of effective measures to support them.

Thus, regarding the availability of the necessary infrastructure, two students reported it as being “very bad” and 4 students reported it as being “bad” in terms of the possibility to connect to the Internet and the availability of digital equipment. Obviously, these students did not have the opportunity to use the online platforms, as they declared. Although most of the students (≈90%) indicated that they have the necessary infrastructure, the next two items show that communication with teachers was not so effective. We believe that even the neutral responses betray actually a lack of communication. On one hand, this “short circuit” felt in the teacher-student communication may be caused by the students’ lack of learning motivation, that, as the results show, obtained the lowest score. On the other hand, the teachers’ lack of experience in online education can be another explanation. The latter idea is somehow supported by the students’ answers to the item regarding the quality of online learning content; most probably teachers did not have the necessary time (everything was rapidly done) and maybe, some of them, not even the skill to design the most appropriate online learning content to keep students “engaged” in front of the screen. In the matter of online assessment/examination, students were mostly satisfied (≈83%), according to their answers to item 8 in Table 3. However, 12.59% of them showed a neutral position which can either betray a slight dissatisfaction with the earned grades/examination method or reflects an objective analysis of their own performance and lack of involvement (something like: “I received what I deserved”; such an example is offered by an engineering student who ranked with “very good” all the items, except the “motivation to learn” and the “assessment/examination”, which he ranked as “very bad”).

As for the tools that students found suitable for online learning (question 3), they mentioned online platforms, e.g., Microsoft Teams platform, implemented at institutional level, but also other platforms such as Zoom, Google Classroom, Edmodo, Cisco Webex, Skype; social networks; YouTube (very useful to find videos for different laboratories, as students stated), applications–WhatsApp.

**Figure 2:**
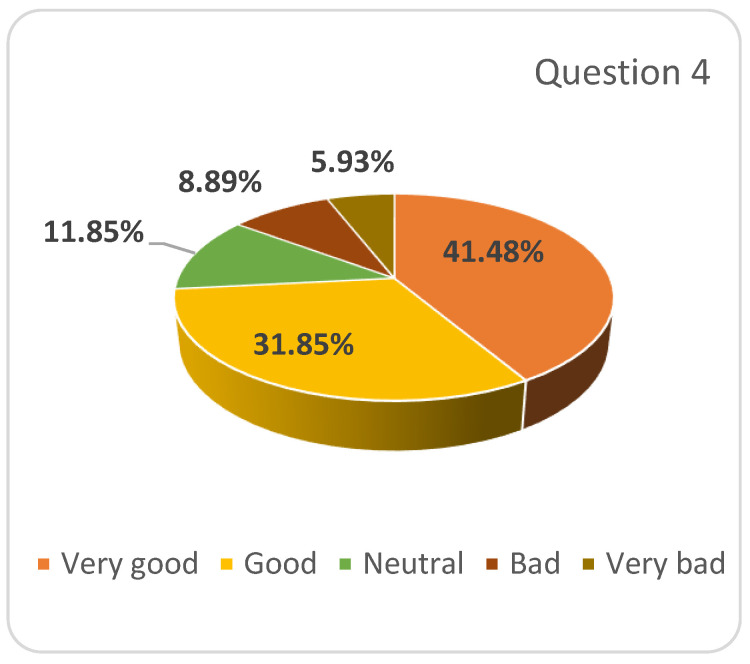
Respondents’ answers to question 4: One option for the development of the educational process in the academic year 2020–2021 is to combine traditional (face-to-face) education with online education. What do you think about this option, considering the learning needs of students?

The next issue addressed in the survey was the possibility of combining traditional education with online education in the academic year 2020–2021 (question 4). When asked how they find this option considering their learning needs, most of the students (73.33%) agreed with it, 11.85% expressed a neutral position while 14.81% of them found it as a “bad” and “very bad idea” (Figure 2).

**Figure 3:**
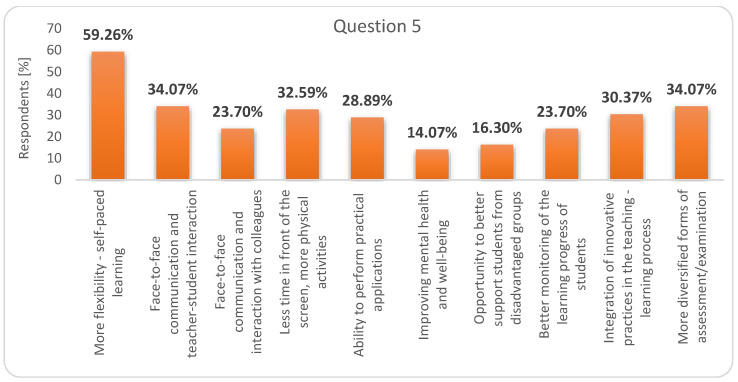
Respondents’ answers to question 5: What would be the advantages of combining face-to-face education with online education?

Among the advantages of combining the two forms of education (question 5), they put “more flexibility-self-paced learning” first, in the second place, in equality, “face-to-face communication and teacher-student interaction” and “more diversified forms of assessment/examination” and in third place, “less time in front of the screen, more physical activities” (Figure 3).

**Figure 4:**
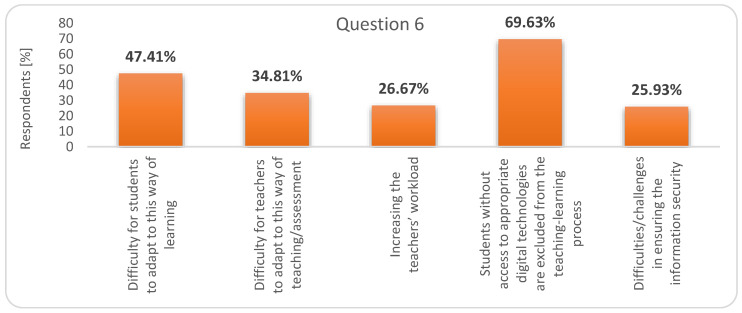
Respondents’ answers to question 6: What would be the disadvantages of combining face-to-face education with online education?

Among the disadvantages of combining the two forms of education (question 6), students ranked in first position “Students without access to appropriate digital technologies are excluded from the teaching-learning process”, thus showing an empathetic attitude with their colleagues, even if those who answered, mostly, have the necessary means. In second place was ranked, “Difficulty for students to adapt to this way of learning” and in third place, “Difficulty for teachers to adapt to this way of teaching/assessment” (Figure 4). 

**Figure 5:**
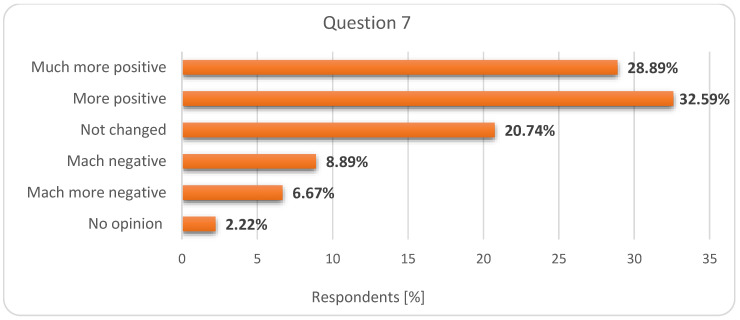
Respondents’ answers to question 7: What is your opinion on the online education considering the experience during the COVID-19 pandemic?

By analysing these results as a whole, we see that students want more flexibility in the teaching–learning–assessment process (which online education can provide) but they are aware of the importance of teacher-student face-to-face interaction and communication and the difficulties that can be encountered online, both in terms of infrastructure and skills. Consequently, when asked how the COVID-19 experience impacted their opinion on online education (question 7), 28.89% of students stated that it became “much more positive”, 32.59% that it became “much positive”, for 20.74% it did not change and for 15.56% got “much negative”/“much more negative”. 2.22% of students did not express their opinion (Figure 5). 

**Figure 6:**
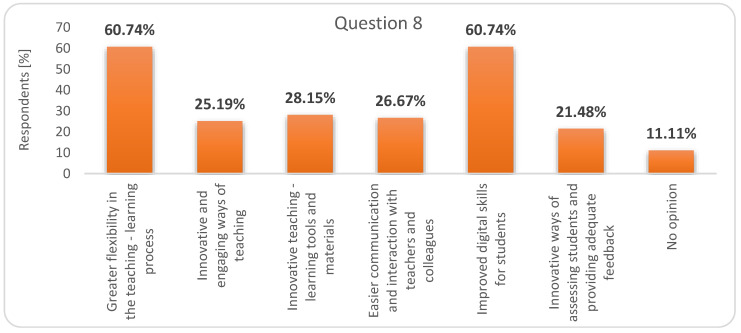
Respondents’ answers to question 8: What would be the main advantages of online education in the future?

When asked about the benefits of online education in the future (question 8), 60.74% of students considered that it offers “greater flexibility in the teaching - learning process” and contributes to the development of their digital skills. Then, 28.15% of students ranked the “innovative teaching-learning tools and materials” that could be used in the teaching-learning process (Figure 6). 

**Figure 7:**
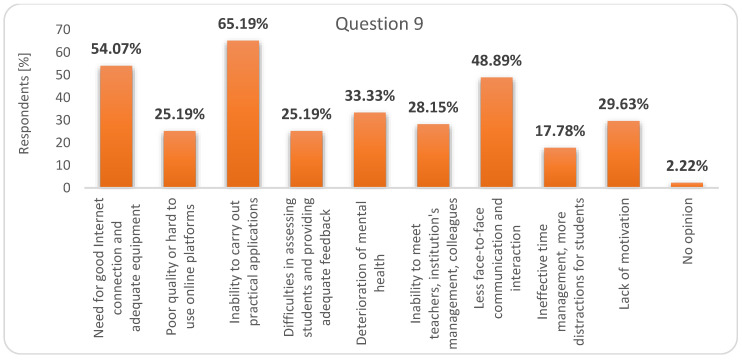
Respondents’ answers to question 9: What would be the main disadvantages of online education in the future?

But the great disadvantage of digital education (question 9) would be the “inability to carry out practical applications” (Figure 7). This is it. Due to the specifics of the two faculties, many disciplines are provided with practical applications. Students depend on access to laboratories to develop key skills and competences. Those from the Faculty of Sport need dedicated training rooms/sport fields, access to medical equipment, access to patients (alleged or real, in profile clinics). Engineering students need technological equipment, tools and devices, models, machine-tools etc. Practical applications cannot be replaced by online learning [11,29].

**Figure 8:**
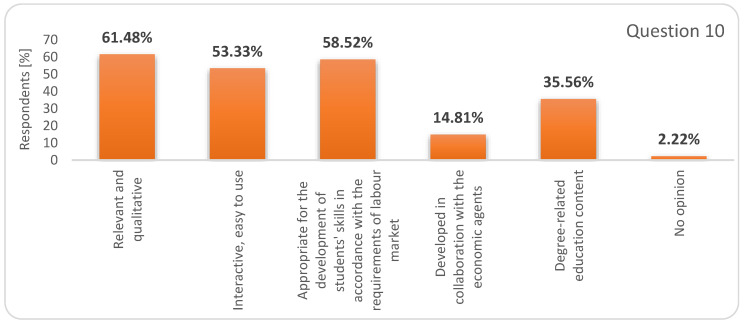
Respondents’ answers to question 10: What makes the online learning resources and content useful?

Regarding the educational resources/contents for online learning, (question 10 in the survey), we can notice that students have very realistic and increased expectations related to these materials; firstly, these must be relevant and qualitative, then develop skills directly linked to the needs of the labour market, and thirdly, they should be interactive and easy to use (Figure 8).

In order to determine what students liked about the online assessment/examination, their answers to the open-ended question on this topic were analysed (question 11).

Thus, 32.58% of students mentioned the lack of stress/comfort related to the examination; 26.66% of students highlighted a higher flexibility (the possibility to take the exam in other locations—e.g., at work, for those who have a job, without having to miss work); 5.18% of students emphasized the faster examination compared to face-to-face examination; 4.44% of them reported a more adequate degree of complexity of the examination requirements/easier examination; 4.44% outlined the improvement of digital skills. Other mentioned benefits were, for instance, the innovative forms of assessment/examination, and even the possibility to cheat during the exams (2 students reported this fact). 11.11% of students stated that there are no advantages of online examination and some of them did not express their opinion. As for the open-ended question regarding the disadvantages of online assessment/examination (question 12), students mentioned the following issues: the lack of Internet connection (18.52%) and adequate digital equipment (3.7%), the possibility to cheat (11.85%), the lack of face-to-face teacher-student interaction (11.85%), the lack of practical applications (9.63%)-in this respect, it is worth mentioning that in face-to-face learning both engineering and sports students are evaluated during the semester at practical applications; probably some of them liked this assessment method and lacked it online. Other reported disadvantages include the subjective evaluation (7.41%), not enough examination time (3.7%), too much time spent in front of the computer (2.22%), lack of motivation (0.74%), insecure platforms (0.74%), stress (0.74%) etc. 11.11% of students stated that there are no disadvantages.

## 4. Discussion

The carried-out survey revealed a series of negative aspects related to the development of online educational processes at UBc from a student’s perspective: (1) There were few students who did not have the necessary infrastructure (e.g., digital equipment, internet connection) to ensure the smooth running of the teaching/learning process; (2) even if the infrastructure existed for most of the students, the communication with teachers was not so effective; (3) the lack of possibility to perform practical applications; (4) lack of some students’ motivation for learning; (5) ineffective online examination (e.g., possibility of cheating, subjective evaluation); (6) lack of socialization/face-to-face interaction and communication of students with teachers and colleagues; (7) potential affecting of mental and physical health (e.g., installation of sedentarism, a problem reported by many other students [10,30]). 

To address these findings and improve the overall quality of the online educational process, at the beginning of the academic year 2020–2021, in the context of COVID-19 pandemic persistence, the university management adopted a series of measures, as follows:

1. Year tutors (one for each study program) had the task of identifying all students who complained about lack of internet, problems with internet connection and lack of appropriate digital equipment. In view of these, they received from faculty secretaries, the contact data of students (telephone and email) and each student was called to confirm the email address and to verify the possibility of connecting to the Microsoft Teams platform. For students who did not have the necessary digital equipment, the university committed to providing them (e.g., tablets) to facilitate their access to classes. However, the problem could be partially solved because the students’ family conditions (e.g., number of people living in the same household, lack of private space, involvement in household chores) may limit their accessibility to online learning [21,31]. For those students who live in areas not covered by internet networks or with a weak internet signal, the university can offer rooms in dormitories with better internet connection. The number of places in dormitories, however, are limited due to the restrictions imposed by the common order of the Minister of Education and Research and the Minister of Health, Order 5487/1494/2020 for the approval of the measures for organizing the activity within the educational units/institutions in epidemiological safety conditions for the prevention of SARS-CoV-2 virus diseases, published in the Official Gazette, Part I no. 804 of 1 September 2020 [32].
2. In order to improve the communication of teachers with students, it was decided in AC and then conveyed at lower hierarchical levels (through faculty councils and department heads) that each teacher would have the obligation to work closely with students, according to the UBc’s slogan: “The university is beside you”. The responsibility was passed to the department heads who must work closely with colleagues in order to make them aware and responsible in getting better engagement with students (e.g., providing fast and appropriate feedback, offering constructive guidance, etc.).
3. To compensate for the lack of physical presence in laboratories, during September 2020, with the help of a local television network, videos were recorded with each teacher briefly describing the laboratory work and the corresponding equipment. These videos will be used during the online meetings with students; we consider that, as high level education providers, universities must pay the utmost attention to the practical training of students because otherwise both the individual and society may pay the price of education failure. On the one hand, students must develop skills that facilitate their access to the labor market, which is still quite challenging for young graduates. On the other hand, employers globally will be facing difficulties in finding workers with proper skills. Thus, ensuring skills is a key factor in developing a sustainable society.
4. A delicate issue that we must address is how to increase students’ motivation in online learning. The phenomenon has been reported by other authors [21,31] and, according to specialists, the key is a careful instructional design and planning of the online educational process. The engagement of students in online classes must be gained by designing engaging tasks and applying a different pedagogy so that to capture the students’ interest [16]. Engagement is the heart of successful learning. According to the specialty literature, the focus must be on both, behavioural and emotional engagement: from paying minimal attention to actively processing the information (e.g., making connections to the previously learned material, critically analysing new information), from being minimally interested in feeling excited and enthusiastic [33,34]. Teachers’ engagement, their sensitivity and interest in students’ development, clearly communicated expectations, respectful and fair treatment of students, are prerequisite for engaging students [35,36,37]. It is not enough to produce page turners that are just screens of text, but to use different tools and methods to draw attention, increase motivation and promote learning–learning for life because almost everything we do requires the use of knowledge in some way, not just having it. However, the educational process is a relationship and in order to be successful, both “parties” need to be equally involved. If students attend classes just for the sake of being present or to get good grades, they are less likely to become engaged beyond a superficial (just get it done) level. In this case, even the best teachers and learning content might not determine a deep engagement of students. This distinction between the two types of engagement is very important. Still, teachers must not give up. According to specialists [38], learners are not necessarily dependent on their will to develop interest or be interested. They may be dependent largely on supports to find ways to connect with the content that they are to learn, and while they need to make their own connections, they are also likely to need support to perceive them. While learners may make a cognitive evaluation about some content, they may also not be aware that their interest has been triggered until much later in the process of its development. As interest develops and deepens, the desire for knowledge and value develop concurrently. In later phases of interest development, they can be so engrossed in engagement that they are not reflecting on it. Similar ideas are promoted by the specialists of Tress Academic, who claim that activity and action induce motivation. According to them, low motivation is not a pre-existing emotional state but a consequence of a lack of activity and explain this by what they call the “motivation spiral”: the more an individual is involved in a certain activity and makes progress in achieving the proposed goals, the more that person becomes satisfied with each achievement, increasing self-confidence, happiness, that further increases the willingness and motivation to work more. In other words, the more you accomplish, the more motivated you feel [39]. Hence, to succeed in the online teaching-learning approach, the crucial elements are: (1) to make sure that students are active not passive learners in front of the screens, and (2) to establish a close teacher–student relationship, based on availability, friendliness and helpfulness, as it influences students’ motivation to learn in a positive way [40,41]. However, the problem for many teachers in Romania (where face-to-face teaching is the main form of education) is “how to do this”, since no governmental program has been implemented to provide teachers with competences and skills for online teaching, which would ensure high quality digital academic experience. Except for various courses focused on the application of tools for interactive education (e.g., the use of digital platforms), nothing else was done. Adopting an online learning environment is not only a technical issue but a pedagogical and instructional challenge [24] that must be overpassed by implementing appropriate instructional strategies [42]. A technical solution that was adopted by the UBc university at the beginning of this academic year, in order to increase student engagement, was to equip classrooms and laboratories with video cameras focused on blackboards, so that teachers could perform various demonstrations and solve problems (e.g., for mathematics) that students could view in real time, as if they were present in class.
5. To ensure the objectivity of assessments and examination to prevent fraudulence and plagiarism, the accent should be placed on continuous evaluation throughout the semester (e.g., by designing homework with a certain degree of complexity). Another possibility would be the open-book online examination [43].
6. For the lack of socialization and face-to-face interaction and communication of students with teachers, there is nothing to do at the moment, as the University is constrained by the measures taken at the national and local levels to limit the spread of the coronavirus. For example, Bacau city is under the red code due to the large number of infested people. However, socialization cannot be neglected; respondents put it in 3rd place in the disadvantages’ hierarchy of online education, after the inability to carry out practical applications and the need for adequate infrastructure. As specialists from University of Oxford stated [44], the university is not just about taking classes; place is also important, as is the relationship with colleagues and teachers [11]. Under these circumstances, a solution would be to engage students and teachers in various extracurricular projects organized by the student league, which is well represented in the university and at the country level. An example that such things could work is presented in [17] (e.g., virtual chairman’s lunches with the organization members).

We believe that each padlock has a key and that there are no problems for which a solution cannot be found, especially if we are open to collaborate and share examples of good practices. Among the countless evils it has caused, the COVID-19 pandemic could have a silver lining, if we know how to exploit it: that of unifying us in a common front to overcome all obstacles put in front of us. However, it is worth mentioning that UBc, as a state public university, has a limited autonomy, so the majority of its measures must fall within the limits allowed by the legislation and regulations applicable at the national level.

### Limitations and Future Directions

The present study has certain limitations. First, it was not intended to be an actual research carried out by specialists in the field of education sciences, but it was an analysis carried out by engineers (the authors are teachers from the Engineering Faculty, some of them members of the top management of the university), with the main stated aim of getting feedback from students in order to identify solutions for removing the reported negative aspects, as well as the opportunities improving the quality of the online educational process in the context of the COVID-19 pandemic. Second, the survey was conducted during the summer holiday, which is why the number of respondents was quite small and they may not be representative for the entire student population. Third, the survey instrument was not a standardized questionnaire. Fourth, the effectiveness of the implemented measures could not be validated at present, but it will be validated in time. For this reason, we intend to conduct new surveys, during this semester, with the support of the Professional Counselling Department, addressing more specific issues related to the students’ performance and well-being in the conditions of participating in crisis education. 

## 5. Conclusions

The conducted study revealed that although the university has taken important steps to ensure the continuity of the educational process in the context of the COVID-19 pandemic, there is still a long way until we have successfully implemented a real and effective online educational system. The challenge to which UBc must face is not related to the technical feasibility of implementing such a system (where things are already quite advanced), but especially to the preparation of human resources for this form of education.

## Figures and Tables

**Table 1:** Comparative analysis of online platforms.

Platform	Recording	Max. Participants Free/Full	Privacy, Security, E2E Encryption	Pricing	Exclusive Feature	Whiteboard
Skype	Storage 30 days in cloud	50 free version	Chats, calls and videos are encrypted, E2E encryption	5–12.5 $/month	Skype to Skype calls; Calls to mobiles and landlines; Group calls; Skype Number; Caller ID; One-to-one video calls; Group video calls; Video messaging; Instant messaging; Send texts (SMS); Send files; Skype Video Conference; Skype Classroom	No
Microsoft Teams	Storage 30 days in cloud	250/250	Microsoft Teams is ISO 270001 and SSAE16 SOC certified	Starting at 5 $/month	Integrated with Office 365, App Integrations; Live Collaboration in Real-Time; Conversation Threads; Collaboration with Clients vendors & Suppliers; One Note; OneDrive	Yes
Zoom	Up to 1 GB of cloud reporting	100/500	Only features with the latest 5.0 have E2E encryption	15–20 $/month	Zoom Chat; Zoom Classroom; Zoom Video Recordings; Zoom Webinars; Google Drive; Hip chat; Dropbox; Slack; HubSpot; Infusionsoft	Yes
Cisco Webex Meetings	Yes, only in own computer	200 participants in the 30-day free trial	E2E encryption Multilayer Security Model Cisco Webex privacy	13.5–26.95 $/month	High-definition (HD) video & audio; Screen & Document Sharing; In-meeting & Recording Notifications Microsoft Office; Google Calendar; Salesforce; Jira; SharePoint Online; OneDrive for Business	Yes
Google Meet	Yes, in Google Drive	100/100	Privacy and security, but no 2E2 encryption	6–25 $/month	Gmail Business email; Meet Video and voice conferencing; Chat Team messaging; Calendar Shared calendars; Drive cloud storage; Docs Word processing; Sheets Spreadsheets; Slides Presentation builder; Forms Professional surveys builder.	No

**Table 2:** The respondents’ affiliation.

Faculty		1st Year	2nd Year	3rd Year	4th Year	Master’s Degree, 1st Year	Master’s Degree, 2nd Year
	Year						
FE		18	29	26	21	2	3
FPSE		9	21	6	-	-	-

**Table 3:** Respondents’ answers to the items regarding question 2: How do you appreciate the online teaching–learning–assessment experience during the COVID-19 pandemic?

Items for Quantifying the Online Experience	Very Good	Good	Neutral	Bad	Very Bad
Possibility to connect to the Internet	70	52	9	2	2
Availability of digital equipment (phone/tablet/laptop/computer)	70	52	7	4	2
Availability and utility/efficiency of the online platforms	72	49	8	4	2
Interaction and communication with teachers (teaching courses, conducting laboratories/seminars/other practical applications)	63	50	14	4	4
Interaction and communication with teachers (providing personalized or group feedback, guidance/tutoring)	58	54	15	5	3
Quality of online learning content (e.g., courses, multimedia content: audio, audio–video, etc.)	62	53	13	5	2
Motivation to learn	40	67	13	7	8
Assessment/Examination	66	46	17	3	3

## Supplementary Materials

The following are available online at https://www.mdpi.com/1660-4601/17/21/7770/s1, The questionnaire.
