# Erratum: Radu, M.-C., et al. The Impact of the COVID-19 Pandemic on the Quality of Educational Process: A Student Survey. *Int. J. Environ. Res. Public Health* 2020, *17*, 7770

**DOI:** 10.3390/ijerph17238778

**Published:** 2020-11-26

**Authors:** Maria-Crina Radu, Carol Schnakovszky, Eugen Herghelegiu, Vlad-Andrei Ciubotariu, Ion Cristea

**Affiliations:** Department of Industrial System Engineering and Management, Faculty of Engineering, “Vasile Alecsandri” University of Bacau, Calea Marasesti 157, 600115 Bacau, Romania; scarol@ub.ro (C.S.); eugen.herghelegiu@ub.ro (E.H.); vlad.ciubotariu@ub.ro (V.-A.C.); icristea@ub.ro (I.C.)

In the original version of our article [1], insufficient acknowledgment was given. We apologize for the original error. To correct this oversight, the acknowledgments section has been altered to more appropriately recognize support and funding.

The corrected acknowledgments are provided below:
