# Supplementary material for: The Impact of the COVID-19 Pandemic on the Quality of Educational Process: A Student Survey
Source: Int J Environ Res Public Health. 2020 Oct 23;17(21):7770. doi: 10.3390/ijerph17217770 (PMC7660608; doi:10.3390/ijerph17217770)
Supplement: Supplementary file 1 [file ijerph-17-07770-s001.pdf]

International Journal of
Environmental Research and Public Health

Supplemental Content

QUESTIONNAIRE

1. To what extent do you consider that the measures taken by "Vasile Alecsandri" University of Bacau during the COVID-19 pandemic to ensure the continuity of the educational process (teaching–learning–assessment) were sufficient and effective:

To a very large extent	To a large extent	To a small extent	To a very small extent	Totally inefficient	No opinion

2. How do you appreciate the online teaching–learning–assessment experience during the COVID-19 pandemic?

	Very good	Good	Neutral	Bad	Very bad
Possibility to connect to the Internet					
Availability of digital equipment (phone/ tablet/ laptop/ computer)					
Availability and utility/efficiency of the online platforms					
Interaction and communication with teachers (teaching courses, conducting laboratories/ seminars/ other practical applications)					
Interaction and communication with teachers (providing personalized or group feedback, guidance/ tutoring)					
Quality of online learning content (e.g. courses, multimedia content: audio, audio-video, etc.)					
Motivation to learn					
Assessment/Examination					

3. Can you give examples of tools that you found particularly useful for the online learning process, including digital platforms, free online courses, etc.?
………

4. One option for the development of the educational process in the academic year 2020-2021 is to combine traditional (face-to-face) education with online education. What do you think about this option, considering the learning needs of students?

Very good	Good	Neutral	Bad	Very bad

5. What would be the advantages of combining face-to-face education with online education?
(please select the relevant alternatives)

€ More flexibility - self-paced learning;
€ Face-to-face communication and teacher-student interaction;
€ Face-to-face communication and interaction with colleagues;

International Journal of
Environmental Research and Public Health

€ Less time in front of the screen, more physical activities;
€ Ability to perform practical applications;
€ Improving mental health and well-being;
€ Opportunity to better support students from disadvantaged groups;
€ Better monitoring of the learning progress of students;
€ Integration of innovative practices in the teaching - learning process;
€ More diversified forms of assessment/examination;
€ Other (please specify) ....

6. What would be the disadvantages of combining face-to-face education with online education?
(please select the relevant alternatives)
€ Difficulty for students to adapt to this way of learning;
€ Difficulty for teachers to adapt to this way of teaching/assessment;
€ Increasing the teachers' workload;
€ Students without access to appropriate digital technologies are excluded from the teaching - learning process;
€ Difficulties/challenges in ensuring the information security;
€ Other (please specify) ....

7. What is your opinion on the online education considering the experience during the COVID-19 pandemic?

Much more positive	More positive	Not changed	Much negative	Much more negative	No opinion

8. ‹What would be the main advantages of online education in the future?
(please select up to 3 options)
€ Greater flexibility in the teaching - learning process;
€ Innovative and engaging ways of teaching;
€ Innovative teaching - learning tools and materials;
€ Easier communication and interaction with teachers and colleagues;
€ Improved digital skills for students;
€ Innovative ways of assessing students and providing adequate feedback;
€ No opinion;
€ Other (please state below) ....

9. What would be the main disadvantages of online education in the future?
(please select up to 3 options)
€ Need for good Internet connection and adequate equipment for students;
€ Poor quality or hard to use online platforms;
€ Inability to carry out practical applications;
€ Difficulties in assessing students and providing adequate feedback;
€ Deterioration of mental health;
€ Inability to meet teachers, institution's management, colleagues;
€ Less face-to-face communication and interaction;

International Journal of
Environmental Research and Public Health

MDPI

€ Ineffective time management, more distractions for students;
€ Lack of motivation;
€ No opinion;
€ Other (please state below) ....

10. What makes the online learning resources and content useful?›
€ Relevant and qualitative;
€ Interactive, easy to use;
€ Appropriate for the development of students' skills in accordance with the requirements of labour market;
€ Developed in collaboration with the economic agents;
€ Degree-related education content;
€ No opinion;
€ Other (please state below) ....

11. In your opinion, what are the advantages of online assessment/examination?
....

12. In your opinion, what are the disadvantages of online assessment/examination?
....

Supplementary data:

Faculty:
€ Faculty of Engineering;
€ Faculty of Physical and Sports Education.

Study programme (optional)
....

Degree:
€ Bachelor’s degree;
€ Master's degree.

Year of study:
€ First year;
€ Second year;
€ Third year;
€ Fourth year.
